# Evaluating the Impact of Preoperative Motor Response and Dural Opening Timing on Outcome Prediction in Patients With Severe Brain Injuries and Intradural Pathologies: An Observational Study

**DOI:** 10.7759/cureus.93671

**Published:** 2025-10-01

**Authors:** A Sathia Prabhu, Kapil Patil

**Affiliations:** 1 Department of Neurosurgery, Jawaharlal Institute of Postgraduate Medical Education and Research, Puducherry, IND; 2 Department of Neurosurgery, Shree Vighnaharta Superspeciality Hospital, Dhule, IND

**Keywords:** dural opening time, gcs, longitudinal cohort study, low- and middle-income countries, mscore, traumatic brain injury

## Abstract

Background

Traumatic brain injury (TBI) is a leading cause of death and disability, particularly in low- and middle-income countries. While the Glasgow Coma Scale (GCS) is widely used for prognostication, its utility is limited in certain scenarios. This study evaluates the prognostic accuracy of the preoperative motor score (Mscore) versus total GCS and explores the impact of surgical timing on outcomes.

Methods

In a prospective cohort study at a tertiary care center, 86 adults (GCS ≤8) undergoing surgery for intradural pathologies were followed. Preoperative clinical parameters, surgical timings (including dural opening time), and radiological findings were recorded. At three months, outcomes were assessed using the Glasgow Outcome Scale-Extended (GOS-E). Statistical analyses included receiver operating characteristic (ROC) curve comparison and multivariable logistic regression.

Results

Among 86 patients, good recovery was achieved in 18.6% of patients, while 51.2% had poor outcomes (death/vegetative state). Higher preadmission and preoperative GCS and Mscores were associated with better outcomes (p < 0.005). ROC curve analysis showed similar predictive ability of Mscore (area under the curve (AUC) 0.68 (95% CI: 0.54-0.82)) and GCS (AUC 0.69 (95% CI: 0.54-0.84)). No single predictor reached statistical significance in multivariable analysis, though trends suggested age and delayed dural opening were associated with poorer outcomes.

Conclusion

The preoperative Mscore offers comparable prognostic value to total GCS in severe TBI. Earlier surgical intervention and proactive airway management may improve outcomes. Further studies with larger samples are needed to confirm these associations.

## Introduction

Head trauma remains a leading cause of mortality and morbidity worldwide, with motor vehicle collisions constituting the primary contributing factor [1,2]. Low- and middle-income countries (LMICs) bear a disproportionate burden, accounting for nearly 85% of all head injuries globally [3]. India reports one of the highest accident rates, with approximately 35 incidents per 1,000 vehicles [4]. A 2005 study estimated that road traffic accidents (RTAs) in India led to around 110,000 deaths, 2.5 million hospitalizations, 8-9 million minor injuries, and economic losses equivalent to 3% of the national gross domestic product (GDP) [5].

Postoperative mortality (POM) following intracranial surgeries exhibits significant regional variability, particularly among LMICs. Reported POM rates range from 2.5% to 39.1% in Africa, 3.6% to 34.8% in Asia, and 1.3% to 12% in Latin America and the Caribbean. These rates are consistently higher than those observed in high-income countries, primarily due to delays in diagnosis and definitive neurosurgical care [6].

Despite guidelines from the Brain Trauma Foundation, management approaches for severe and critical head injuries vary across neurosurgical centers. The Glasgow Coma Scale (GCS) is widely used to assess neurological status; however, its utility is limited in intubated patients, those under the influence of alcohol, or those with facial injuries. In such cases, the motor component of the GCS (motor score (Mscore)) has been shown to be a more reliable and practical prognostic indicator [7,8]. Timely evacuation of subdural hematomas significantly reduces mortality and improves functional outcomes. Mortality rates have been reported as 73% for patients evacuated within two hours of trauma, compared to 94% for those evacuated after four hours [9,10].

However, a 2006 meta-analysis cautioned against a simplistic interpretation of this association, noting that patients operated on earlier often have more severe injuries, confounding the impact of timing alone [11]. More recent meta-epidemiological evidence reinforced these disparities, reporting a pooled traumatic brain injury (TBI)-related mortality of 16.7% (95% CI: 13.7-20.3%) across 31 LMICs and concluding that mortality is three- to four-fold higher than in high-income countries [12].

The present study aimed to evaluate the clinical, radiological, and surgical characteristics of patients with severe TBI and intradural pathologies (acute subdural hematoma, cerebral contusion with mass effect, and traumatic subarachnoid hemorrhage requiring surgical decompression). Specifically, we investigated the prognostic value of the preoperative Mscore compared with the overall GCS score and examined the relationship between dural opening time (as opposed to skin incision time) and patient outcomes.

## Materials and methods

Study design and participant selection

This prospective longitudinal cohort study was conducted at Jawaharlal Institute of Postgraduate Medical Education and Research (JIPMER), Puducherry, India, after the study was approved by the institute's Institutional Ethics Committee (approval number: JIP/IEC/2017/0098) and included adult patients aged 18-70 years who had sustained severe brain injuries, defined by a GCS score of 8 or below. All eligible patients underwent surgical intervention for intradural pathologies, which included emergency neurosurgical procedures (craniotomy or decompressive craniectomy) performed at the discretion of the attending neurosurgeon based on intraoperative findings. To ensure cohort homogeneity and minimize confounding factors, patients were excluded if they exhibited absent brainstem reflexes, had pre-existing neurological disorders, sustained multiple traumatic injuries, presented with purely extradural pathology, were on anticoagulant therapy, died due to systemic diseases unrelated to the primary pathology, suffered penetrating injuries with gross contamination, exhibited significant vascular trauma, or developed culture-positive postoperative meningitis.

Sample size and data collection

A total of 86 participants were enrolled over an 18-month period (May 6, 2017-October 19, 2018), based on an anticipated in-hospital mortality rate of approximately 15% [12]. The minimum required sample size was calculated using the single-proportion formula $\text{n}=\left( \text{Z}^{2} \times\text{p}\times\left( 1-\text{p}\text{} \right)\right)/\text{d}^{2}$, where Z = 1.96 (95% confidence), p = 0.17 (expected severe TBI mortality in LMICs, derived from a meta-epidemiological study reporting a pooled mortality of 16.7%, 95% confidence interval (CI) 13.7-20.3%), and d = 0.08 (absolute precision), yielding an estimated sample size of 84. To account for potential exclusions and loss to follow-up, 86 patients were ultimately recruited. Baseline demographic and clinical variables were recorded, including age, sex, mechanism of injury, and pre-existing comorbidities, while preoperative assessments comprised GCS scores with emphasis on the Mscore, timing to dural opening, duration on ventilator, and computed tomography (CT) findings interpreted by institutional radiologists who were not blinded. Neurological evaluations (GCS and Mscore) were performed by neurosurgery residents trained in standardized protocols. Intraoperative findings and postoperative events were documented, including complications, length of hospital stay, in-hospital mortality, and GCS at discharge or on postoperative day 5, whichever occurred earlier, while long-term outcomes were assessed through outpatient visits or structured telephonic interviews.

Measurement tools and standardization

All data were collected using standardized protocols to ensure consistency and reproducibility. Neurological status and functional outcomes were evaluated using validated tools, including the GCS and the Glasgow Outcome Scale-Extended (GOS-E). The GOS-E classifies outcomes into eight categories, ranging from death to upper good recovery. For analysis, we consolidated these into three outcome levels: (1) death or vegetative state (GOS-E 1-2), (2) disability (GOS-E 3-6, encompassing severe and moderate disability), and (3) good recovery (GOS-E 7-8) [13]. Additional clinical parameters were systematically documented to assess motor function, neurological progression, and recovery trajectory.

Statistical analysis

All statistical analyses were performed using the R software (version 4.4.0) (R Foundation for Statistical Computing, Vienna, Austria). Continuous variables were summarized as mean ± standard deviation (SD) or median with interquartile range (IQR), depending on the distribution of the data. Categorical variables were presented as frequencies and percentages.

For group comparisons between the two categories (survivors and non-survivors), independent t-tests were used for normally distributed continuous variables, and the Wilcoxon rank-sum test was used for non-normally distributed variables (specifically, age). For comparisons across more than two groups (GOS-E classification), one-way analysis of variance (ANOVA) was used for normally distributed continuous variables, and the Kruskal-Wallis test was used for age. Categorical variables were compared using the chi-squared test.

Receiver operating characteristic (ROC) curve analysis was employed to assess the predictive performance of the preoperative Mscore and total GCS score in relation to postoperative outcomes measured by the GOS-E. The area under the curve (AUC) was calculated for each metric, and DeLong's test was used to compare the AUCs of correlated ROC curves.

Multivariable logistic regression was conducted to identify independent predictors of in-hospital mortality. Clinically relevant variables and those with a univariable p-value <0.05 were included in the model. Results were reported as odds ratios (OR) with corresponding 95% CI. A two-sided p-value of <0.05 was considered statistically significant. Visualizations, including bar and lollipop plots, were used to support data interpretation.

## Results

Participant characteristics

A total of 86 patients with severe TBI were included. The median age was highest in the death/vegetative state group (47 years, IQR: 32.8-58.5), followed by the disabled group (38.5 years, IQR: 27.5-56.3) and the good recovery group (32 years, IQR: 25.3-45.5); however, these differences were not statistically significant (p = 0.132). Females constituted 15.1% of the overall cohort, and gender distribution did not differ significantly across outcome groups (p = 0.510).

**Figure 1:**
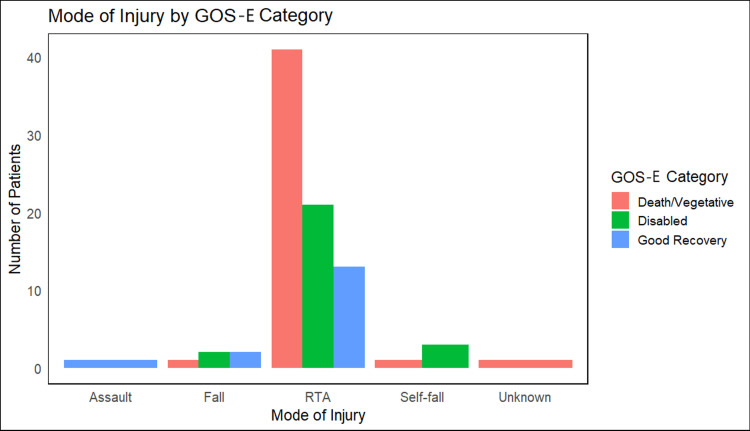
Mode of injury by GOS-E category Bar plot showing the number of participants per injury type. RTA: road traffic accident; GOS-E: Glasgow Outcome Scale-Extended

RTAs were the predominant mechanism of injury in all groups, with no significant variation in injury etiology (p = 0.235) (Figure 1).

Clinical and radiological parameters

Significant differences were observed in preadmission clinical scores across outcome groups. Patients in the good recovery group had higher preadmission GCS (7.06 ± 1.65) compared to the death/vegetative group (5.59 ± 1.72; p = 0.002), along with higher Mscores (Mscore: 4.38 ± 1.26 vs. 3.27 ± 1.39; p = 0.004). These trends were consistent in preoperative assessments (p < 0.005 for both GCS and Mscore). Radiological findings such as basal cistern effacement were significantly more prevalent in the death/vegetative state group (n = 36; 90%) compared with other outcome groups (p = 0.043).

Ventilator duration was longest among patients in the death/vegetative group (mean: 3.89 days), with a statistically significant decreasing trend across better outcome categories (p = 0.006). Both in-hospital and all-cause mortality occurred exclusively within the death/vegetative group (p < 0.001) (Table 1).

**Table 1:** Baseline demographic and clinical characteristics stratified by GOS-E categories at three months Values are presented as N (%) for categorical variables and mean ± SD for continuous variables and median (IQR) for age. Statistical comparisons were performed using the chi-squared test for categorical variables, one-way ANOVA for continuous variables, and the Kruskal-Wallis test for age. For each comparison, the test statistic^#^ (chi-squared value, F value, or Kruskal-Wallis H statistic) and corresponding p-value are reported. A p-value of ≤0.05 was considered statistically significant. RTA: road traffic accident; Mscore: motor score; GCS: Glasgow Coma Scale; GOS-E: Glasgow Outcome Scale-Extended; ANOVA: analysis of variance; IQR: interquartile range

GOS-E parameters	Death/vegetative	Disabled	Good recovery	Statistic^#^	P-value
n	44	26	16	-	-
Age (median (IQR))	47.00 (32.75, 58.50)	38.50 (28.00, 50.75)	32.00 (28.75, 48.00)	4.05	0.132
Female (%)	7 (15.9)	5 (19.2)	1 (6.2)	1.34	0.51
Mode of injury (%)					
Assault	0 (0.0)	0 (0.0)	1 (6.2)	11.97	0.235
Fall	1 (2.3)	2 (7.7)	2 (12.5)		
RTA	41 (93.2)	21 (80.8)	13 (81.2)		
Self-fall	1 (2.3)	3 (11.5)	0 (0.0)		
Unknown	1 (2.3)	0 (0.0)	0 (0.0)		
Preadmission Mscore (mean (SD))	3.27 (1.39)	4.12 (1.07)	4.38 (1.26)	6.06	0.004
Preadmission GCS (mean (SD))	5.59 (1.72)	6.69 (1.44)	7.06 (1.65)	6.54	0.002
Preoperative Mscore (mean (SD))	3.27 (1.34)	4.15 (1.05)	4.38 (1.26)	6.66	0.002
Preoperative GCS (mean (SD))	5.56 (1.61)	6.58 (1.30)	7.19 (1.94)	7.31	0.001
Right pupillary diameter (mm) (mean (SD))	2.40 (0.65)	2.29 (0.53)	2.25 (0.58)	0.47	0.626
Left pupillary diameter (mm) (mean (SD))	2.31 (0.65)	2.21 (0.64)	2.06 (0.44)	0.96	0.389
Intubation	6 (13.6)	5 (19.2)	9 (56.2)	17.64	0.001
Pulse (bpm) (mean (SD))	87.48 (19.26)	87.58 (15.39)	84.56 (16.75)	0.18	0.835
Systolic blood pressure (mmHg) (mean (SD))	122.27 (24.01)	125.54 (13.27)	121.62 (17.35)	0.27	0.764
Diastolic blood pressure (mmHg) (mean (SD))	74.45 (12.82)	76.65 (10.83)	76.00 (10.56)	0.31	0.737
Respiratory rate (breaths/min) (mean (SD))	18.55 (5.29)	16.58 (3.25)	18.44 (5.23)	1.51	0.226
Contusion (%)	9 (20.5)	5 (19.2)	4 (25.0)	0.21	0.9
Subdural hematoma (%)	8 (18.2)	5 (19.2)	4 (25.0)	0.35	0.839
Subarachnoid hemorrhage (%)	29 (65.9)	19 (73.1)	13 (81.2)	1.42	0.491
Basal cistern effacement (%)	36 (90.0)	18 (72.0)	10 (62.5)	6.28	0.043
Midline shift (mm) (mean (SD))	6.59 (4.34)	5.99 (4.59)	4.90 (2.76)	0.97	0.382
Total operative duration (hours) (mean (SD))	3.26 (0.94)	4.46 (6.28)	3.55 (1.13)	0.96	0.388
Time from injury to casualty admission (hours) (mean (SD))	6.83 (11.07)	11.29 (14.40)	11.79 (14.09)	0.29	0.245
Time between casualty admission and surgery (hours) (mean (SD))	10.92 (10.93)	12.12 (8.80)	13.42 (16.87)	0.28	0.752
Time between surgery and dural opening (minutes) (mean (SD))	47.95 (8.58)	46.54 (5.43)	47.19 (8.16)	0.28	0.754
Time between casualty admission and dural opening (hours) (mean (SD))	18.55 (14.85)	24.39 (15.61)	26.00 (19.70)	1.78	0.175
Duration on ventilator (days) (mean (SD))	3.89 (3.00)	2.58 (2.42)	1.56 (1.09)	5.43	0.006
Length of hospital stay (days) (mean (SD))	7.84 (5.02)	7.65 (3.21)	9.94 (10.01)	0.9	0.41
Hospital mortality (%)	25 (56.8)	0 (0.0)	0 (0.0)	33.64	<0.001
All-cause mortality (%)	42 (95.5)	0 (0.0)	0 (0.0)	77.36	<0.001

All-cause mortality analysis

Comparisons between survivors (n = 43) and non-survivors (n = 42) demonstrated that survivors had significantly higher preadmission and preoperative GCS and Mscores (mean Mscore: 4.19 vs. 3.24; GCS: 6.77 vs. 5.57; both p = 0.001). Intubation was more frequently documented among survivors (32.6% vs. 11.9%; p = 0.039); however, this association is likely confounded by baseline neurological status, particularly GCS, and should not be interpreted as a direct causal relationship. Non-survivors required a longer duration of ventilatory support compared with survivors (3.74 ± 2.68 vs. 2.42 ± 2.63 days; p = 0.024) (Table 2).

**Table 2:** Baseline demographic and clinical characteristics stratified by all-cause mortality Values are presented as N (%) for categorical variables and mean ± SD for continuous variables and median (IQR) for age. Statistical comparisons were performed using the chi-squared test for categorical variables, the independent t-test for continuous variables (except age), and the Wilcoxon rank-sum test for age. For each comparison, the test statistic^#^ (chi-squared value, t-value, or W statistic) and corresponding p-value are reported. A p-value of ≤0.05 was considered statistically significant. One patient was lost to follow-up; hence, the outcome analysis included 85 patients. RTA: road traffic accident; Mscore: motor score; GCS: Glasgow Coma Scale; GOS-E: Glasgow Outcome Scale-Extended; IQR: interquartile range

GOS-E parameters	Non-survivors	Survivors	Statistic^#^	P-value
n	42	43	-	-
Age (median (IQR))	44.71 (15.06)	39.47 (13.56)	1095.5	0.095
Female (%)	7 (16.7)	5 (11.6)	0.13	0.72
Mode of injury (%)				
Assault	0 (0.0)	1 (2.3)	5.01	0.315
Fall	1 (2.4)	4 (9.3)		
RTA	39 (92.9)	35 (81.4)		
Self-fall	1 (2.4)	3 (7.0)		
Unknown	1 (2.4)	0 (0.0)		
Preadmission Mscore (mean (SD))	3.24 (1.39)	4.19 (1.14)	-3.43	0.001
Preadmission GCS (mean (SD))	5.57 (1.74)	6.77 (1.51)	-3.38	0.001
Preoperative Mscore ≤3	23 (54.8)	10 (23.3)	4.19	0.006
Preoperative GCS ≤5	20 (48.8)	9 (20.9)	6.02	0.014
Right pupillary diameter (mm) (mean (SD))	2.42 (0.66)	2.27 (0.54)	1.14	0.257
Left pupillary diameter (mm) (mean (SD))	2.32 (0.66)	2.15 (0.56)	1.28	0.204
Intubation	5 (11.9)	14 (32.6)	6.5	0.039
Pulse (bpm) (mean (SD))	88.12 (19.25)	86.09 (16.04)	0.53	0.599
Systolic blood pressure (mmHg) (mean (SD))	122.86 (24.43)	123.26 (14.96)	-0.09	0.928
Diastolic blood pressure (mmHg) (mean (SD))	74.43 (13.08)	76.26 (10.52)	-0.71	0.479
Respiratory rate (breaths/min) (mean (SD))	18.48 (5.33)	17.49 (4.21)	0.95	0.345
Contusion (%)	9 (21.4)	9 (20.9)	0	1
Subdural hematoma (%)	8 (19.0)	9 (20.9)	0	1
Subarachnoid hemorrhage (%)	27 (64.3)	33 (76.7)	1.05	0.31
Basal cistern effacement (%)	34 (89.5)	30 (69.8)	3.61	0.06
Midline shift (mm) (mean (SD))	6.57 (4.42)	5.61 (3.97)	1.06	0.294
Total operative duration (hours) (mean (SD))	3.28 (0.96)	4.07 (4.92)	-1.04	0.306
Time from injury to casualty admission (hours) (mean (SD))	6.95 (11.32)	11.15 (13.86)	-0.63	0.131
Time between casualty admission and surgery (hours) (mean (SD))	10.90 (11.18)	12.49 (12.18)	0.67	0.532
Time between surgery and dural opening (minutes) (mean (SD))	47.98 (8.77)	46.86 (6.46)	0.67	0.505
Time between casualty admission and dural opening (hours) (mean (SD))	18.65 (15.20)	24.42 (16.91)	1.06	0.102
Postoperative Mscore ≤3	11 (29.7)	4 (9.3)	7.6	0.041
Duration on ventilator (days) (mean (SD))	3.74 (2.68)	2.42 (2.63)	2.29	0.024
Length of hospital stay (days) (mean (SD))	7.64 (4.87)	8.74 (6.73)	-0.87	0.391

In multivariable logistic regression analysis (Table 3), no single predictor reached statistical significance. However, age demonstrated a trend toward increased mortality risk (OR: 1.03; 95% CI: 1.00-1.08; p = 0.050), while higher preadmission GCS scores showed a non-significant trend toward reduced mortality (OR: 0.60; 95% CI: 0.12-2.90; p = 0.520).

**Table 3:** Multivariable logistic regression analysis for all-cause mortality Mscore: motor score; OR: odds ratio; CI: confidence interval; SE: standard error

Variable	OR	95% CI lower	95% CI upper	SE	P-value
(Intercept)	0.25	0.00	87.22	2.98	0.64
Age	1.04	1.00	1.08	0.02	0.05
Female	1.46	0.33	6.72	0.76	0.62
Preadmission Mscore	3.64	0.16	84.93	1.57	0.41
Preadmission Glasgow Coma Scale	0.60	0.12	2.90	0.81	0.52
Preoperative Mscore	0.30	0.02	6.39	1.49	0.41
Preoperative Mscore ≤3	1.47	0.33	6.04	0.74	0.60
Preoperative Glasgow Coma Scale	0.32	0.06	1.55	0.82	0.17
Intubation	1.00	0.94	1.08	0.03	0.90
Time between surgery and dural opening	1.21	0.96	1.58	0.12	0.13
Duration on ventilator	3.14	0.25	46.92	1.32	0.39
Length of hospital stay	0.91	0.80	1.02	0.06	0.13

Predictors

**Figure 2:**
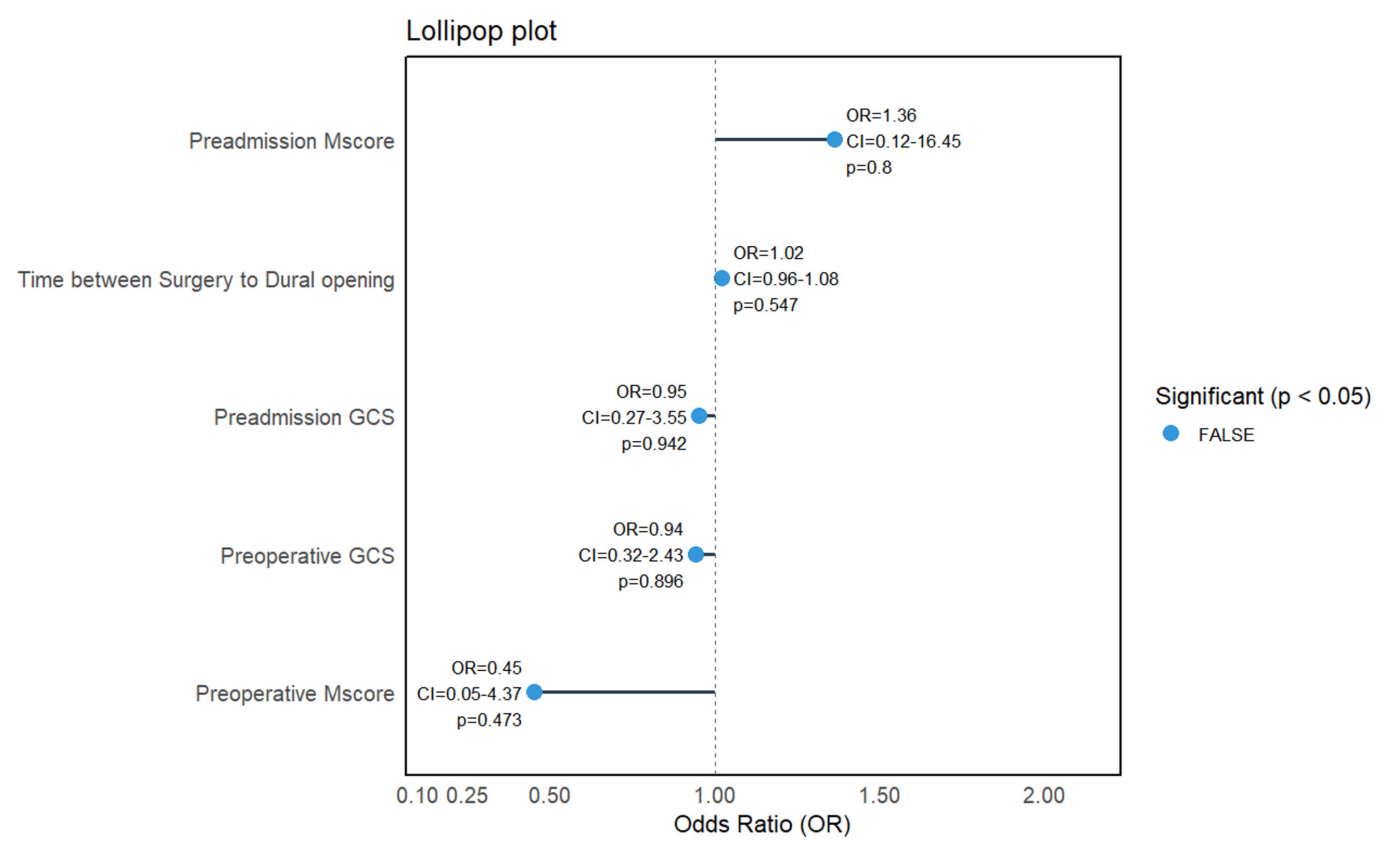
Association of key clinical parameters and dural opening timing with mortality Mscore: motor score; GCS: Glasgow Coma Scale

A lollipop plot (Figure 2) illustrates the direction and magnitude of associations between selected preoperative variables and all-cause mortality. Per-unit increases in preadmission Mscores and longer intervals between surgery initiation and dural opening were associated with increased mortality risk. Conversely, per-unit increases in preadmission and preoperative GCS and Mscores were associated with reduced mortality odds. However, none of these associations achieved statistical significance, indicating that larger sample sizes or further adjustment for confounders may be necessary to validate these trends.

Predictive accuracy of clinical scores

**Figure 3:**
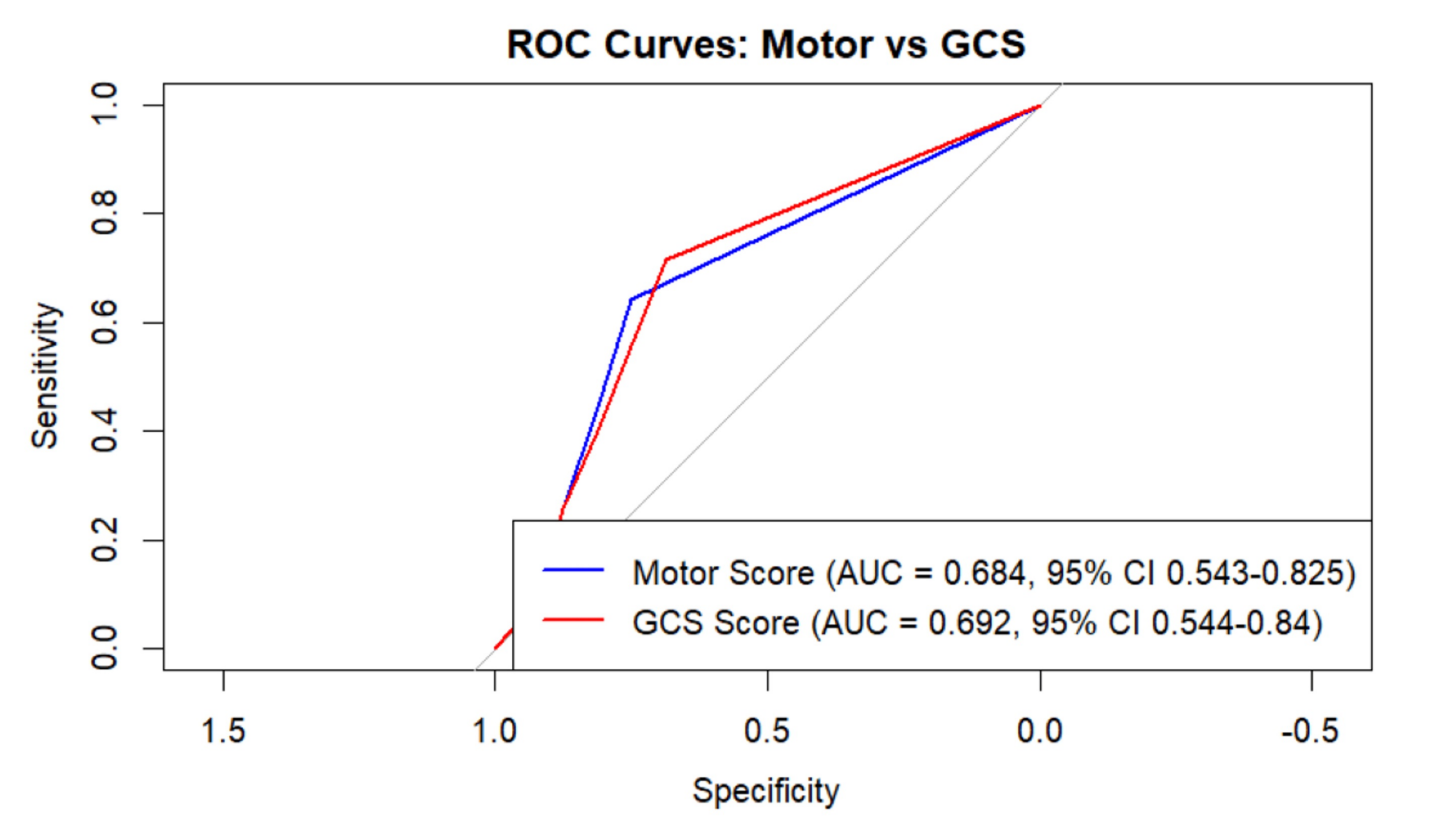
ROC curve: Mscore vs. GCS ROC curve comparing the predictive accuracy of GCS and Mscores for poor outcomes. ROC: receiver operating characteristic; AUC: area under the curve; Mscore: motor score; GCS: Glasgow Coma Scale

ROC curve analysis was conducted to evaluate the prognostic utility of the preoperative Mscore and total GCS score in predicting outcomes as defined by the GOS-E. The AUC for the Mscore was 0.68 (95% CI: 0.54-0.82), compared to 0.69 (95% CI: 0.54-0.84) for the total GCS. Although the GCS showed a numerically higher discriminative ability, DeLong's test found no statistically significant difference between the two (p = 0.496) (Figure 3).

## Discussion

Despite significant advancements in the management of TBI, mortality rates remain unacceptably high. Previous studies have reported mortality rates ranging from 26.7% to 41.4% in Level I trauma centers [14,15], while in LMICs, rates may be as high as 51% compared to 30% in high-income countries [3]. In our cohort, 50% (n = 42) of patients succumbed to their injuries, consistent with outcomes observed in resource-limited settings. Our study aimed to evaluate functional outcomes at three months following severe TBI in adults aged 18-70 years. Outcomes assessed using the GOS-E revealed that 51.2% (n = 44) of patients were either deceased or in a vegetative state (GOS-E ≤2), while only 18.6% (n = 16) achieved good recovery. This distribution aligns with findings by Beck et al., who reported poor outcomes in approximately 70% of patients with severe brain injuries [16].

Injury patterns in our cohort also mirrored global trends. The mean age of 42.22 years parallels findings from South Africa and China, where TBI primarily affects middle-aged adults. RTAs accounted for the majority of injuries (n = 74; 94%), as illustrated in Figure 1. This reflects broader issues in LMICs such as inadequate road safety compliance and increased exposure among working-age males. The underrepresentation of female patients (n = 13; 15.1%) may be due to social and behavioral factors, including lower rates of vehicular usage and alcohol exposure among women. Such demographic and epidemiological parallels highlight the consistency of TBI risk profiles across diverse geographic settings.

The relationship between surgical timing and outcomes remains complex. Although earlier evacuation of mass lesions (e.g., acute subdural hematoma) has been associated with improved survival in selected contexts [9,17], we did not observe a statistically significant association between injury-to-surgery (or dural opening) intervals and mortality. Notably, patients with favorable outcomes showed longer admission-to-surgery intervals; this counterintuitive trend likely reflects confounding by indication: more severely injured patients (lower GCS/Mscore, anisocoria, greater midline shift, basal cistern effacement, large subdural/contusional burden, hemodynamic instability) were triaged for rapid intervention, whereas physiologically stable patients with less mass effect proceeded after resuscitation and optimization. In our center, the clinical factors guiding operative timing included preadmission and preoperative GCS/Mscore, pupillary reactivity/anisocoria, CT markers of mass effect (midline shift magnitude, basal cistern status, presence of subdural hematoma and/or dominant contusion), and overall hemodynamic stability; logistical elements (operating-room availability and parallel resuscitation) also influenced timing but were not systematically captured [18]. From a practical standpoint in LMICs, these bedside neurological and radiological cues can help prioritize scarce operating-room resources toward patients with robust indications for urgent decompression while permitting brief, goal-directed resuscitation in more stable presentations.

Pupillary abnormalities are established markers of herniation risk and poor prognosis due to midbrain compression [19]. In our cohort, basal cistern effacement, an indirect indicator of raised intracranial pressure, was significantly associated with worse outcomes, whereas absolute pupillary size did not differ between outcome groups. Mortality was higher among patients with preoperative GCS ≤5 (20/29; 68.97%) and those with preoperative Mscore ≤3 (23/33; 69.7%) (see Table 2), underscoring the importance of initial neurological severity. In ROC curve analyses, Mscore and total GCS demonstrated similar discrimination; the AUC for GCS was modestly higher, but the difference was not statistically significant. Accordingly, our data support the non-inferiority of Mscore relative to total GCS for prognostication in this surgical severe TBI cohort, rather than the superiority of either measure. Apparent paradoxes in the lollipop plot (e.g., a positive coefficient for preadmission Mscore or for longer time to dural opening) did not achieve statistical significance and likely reflect confounding by indication and limited power.

Limitations

This single-center cohort study with a modest sample size (n = 86) limits statistical power and generalizability. Imaging interpretations were not blinded, and airway management and operative timing were not protocolized, introducing potential selection bias and residual confounding. The retrospective design also meant that some variables were incompletely documented. In particular, detailed timing of prehospital or emergency department intubation and the presence of combined radiological lesions (e.g., subdural hematoma with contusion) were not systematically recorded. In addition, differences in ventilator duration observed across outcome groups likely reflect underlying injury severity rather than a direct causal effect. Variability in postoperative care, including ICU access, ventilatory support strategies, and rehabilitation, may also have influenced patient outcomes. Larger, prospective, multicenter studies with standardized data collection are needed to validate these findings.

## Conclusions

In this cohort of patients from a single LMIC center with severe TBI and intradural pathologies, the preoperative Mscore demonstrated non-inferior prognostic accuracy compared with the total GCS, with both measures showing similar discrimination for mortality and functional outcomes. Since GCS is an established and widely validated tool, our findings highlight the practical value of Mscore as a simpler alternative when a complete GCS assessment is not feasible, such as in intubated patients or those with facial injuries. Although a longer time to dural opening was numerically associated with mortality, this relationship did not reach statistical significance and is likely explained by confounding from injury severity and triage factors. The retrospective single-center design, modest sample size, and variability in perioperative and postoperative care limit the generalizability of these results. Nonetheless, the study provides evidence supporting the integration of both Mscore and GCS into early risk stratification for severe TBI and underscores the need for larger, prospective, multicenter studies to validate these findings.
